# Knockout of serine-rich single-pass membrane protein 1 (*Ssmem1*) causes globozoospermia and sterility in male mice^†^

**DOI:** 10.1093/biolre/ioaa040

**Published:** 2020-04-17

**Authors:** Kaori Nozawa, Qian Zhang, Haruhiko Miyata, Darius J Devlin, Zhifeng Yu, Seiya Oura, Takayuki Koyano, Makoto Matsuyama, Masahito Ikawa, Martin M Matzuk

**Affiliations:** 1 Center for Drug Discovery, Baylor College of Medicine, Houston, TX; 2 Department of Pathology & Immunology, Baylor College of Medicine, Houston, TX; 3 Department of Experimental Genome Research, Research Institute for Microbial Diseases, Osaka University, Suita, Osaka, Japan; 4 Interdepartmental Program in Translational Biology & Molecular Medicine, Baylor College of Medicine, Houston, TX; 5 Graduate School of Pharmaceutical Sciences, Osaka University, Suita, Osaka, Japan; 6 Division of Molecular Genetics, Shigei Medical Research Institute, Okayama, Japan; 7 Graduate School of Medicine, Osaka University, Suita, Osaka, Japan; 8 The Institute of Medical Science, The University of Tokyo, Tokyo, Japan

**Keywords:** sperm, spermatogenesis, fertilization, male infertility, null mutation/knockout

## Abstract

Globozoospermia (sperm with an abnormally round head shape) and asthenozoospermia (defective sperm motility) are known causes of male infertility in human patients. Despite many studies, the molecular details of the globozoospermia etiology are still poorly understood. Serine-rich single-pass membrane protein 1 (*Ssmem1*) is a conserved testis-specific gene in mammals. In this study, we generated *Ssmem1* knockout (KO) mice using the CRISPR/Cas9 system, demonstrated that *Ssmem1* is essential for male fertility in mice, and found that SSMEM1 protein is expressed during spermatogenesis but not in mature sperm. The sterility of the *Ssmem1* KO (null) mice is associated with globozoospermia and loss of sperm motility. To decipher the mechanism causing the phenotype, we analyzed testes with transmission electron microscopy and discovered that *Ssmem1*-disrupted spermatids have abnormal localization of Golgi at steps eight and nine of spermatid development. Immunofluorescence analysis with anti-Golgin-97 to label the trans-Golgi network, also showed delayed movement of the Golgi to the spermatid posterior region, which causes failure of sperm head shaping, disorganization of the cell organelles, and entrapped tails in the cytoplasmic droplet. In summary, SSMEM1 is crucial for intracellular Golgi movement to ensure proper spatiotemporal formation of the sperm head that is required for fertilization. These studies and the pathway in which SSMEM1 functions have implications for human male infertility and identifying potential targets for nonhormonal contraception.

## Teaser

Absence of serine-rich single-pass membrane protein 1 (SSMEM1) leads to male infertility because of globozoospermia in mice.

## Introduction

Spermatozoa transmit the genetic information to the next generation and unique morphological features specialized for fertilization. It is reported that more than 1000 genes are dominantly expressed in human and mouse testes [1, 2]. The deficiency of those genes has been proposed to cause spermatogenesis defects and infertility in human patients [3]. In addition, many testis-enriched genes were reported to be evolutionarily conserved in mammals including mouse and human [4, 5]. The analysis of knockout (KO) mouse models, which carry null gene mutations, is a powerful strategy to study male infertility pathogenesis attributable to the lack of in vitro culture systems of spermatozoa [6].

The success of spermatogenesis is critical for maintenance of male fertility and requires a well-organized process in which spermatogonia undergo mitosis, meiosis, and spermiogenesis. Furthermore, spermiogenesis can be divided into the following main phases: Golgi, acrosome cap/elongation, and maturation phases [7]. The Golgi apparatus produces proacrosomal vesicles during the Golgi phase, which coalesce and result in the acrosomal vesicle adjacent to the nuclear membrane on the opposite side of the tail. The acrosome cap phase is characterized by the spread of the acrosome over the spermatid nucleus, which coincides with tail development [8]. As the spermatid cytoplasm and nucleus elongates, the organelles, including Golgi and mitochondria, move from the acrosome and migrate to the caudal aspect. During the maturation phase, the spermatozoa are fully developed and reshaped. Eventually the mitochondria locate at the midpiece of the tail and the Golgi is discarded into cytoplasmic droplets with other organelles so that spermatozoa obtain the morphology suitable for fertilization [9, 10]. To explain elongation of the nuclei, the current hypothesis is that the manchette, consisting of microtubules and actin filaments, drag and compel the nucleus to become more dense and elongate toward the tail aspect [11].

Globozoospermia, which is a spermiogenesis defect, is one cause of human male infertility [12]. The characteristic feature of globozoospermia is an abnormal nuclear shape and arrangement of the sperm mitochondria. Some genes, such as *DPY19L2, PICK1*, and *SPATA16,* have been found as causative genes in human patients [13, 14]. Human globozoospermic spermatozoa have also shown an incapability of natural fertilization because they are unable to bind the zona pellucida and fuse with oocytes [12]. However, these spermatozoa also show low fertilization rates even after intracytoplasmic sperm injection treatment, which is a standard rescue option for infertile men [15], because of oocyte activation failure [16]. Although studies have shown correlation between globozoospermia and the lack of acrosome, the mechanisms inducing globozoospermia are still not fully appreciated.

In our in silico bioinformatic analyses [17, 18], we identified *Ssmem1* as an evolutionarily conserved and testis-specific gene. Here, we generated *Ssmem1* KO mice and verified its significant role in vivo. We observed that lack of *Ssmem1* in male mice alters spermiogenesis, resulting in globozoospermia and sterility. Our ultrastructural data reveal that absence of SSMEM1 alters transport of the Golgi during spermatid elongation, thereby uncovering a role of SSMEM1 in this process.

## Material and methods

### Ethics statement

Mice were maintained in accordance with National Institutes of Health (NIH) guidelines, and all animal procedures were approved by the Institutional Animal Care and Use Committee (IACUC) at Baylor College of Medicine and Osaka University.

### Animals

B6D2F1 purchased from Japan SLC (Hamamatsu, Shizuoka, Japan) or CLEA Tokyo, B6D2F1 mice were used for generating *Ssmem1* mutant founder mice. In-house hybrid mice (C57BL/6J × 129S5/SvEvBrd) were mated with *Ssmem1* heterozygous (HET) mice to expand the line. For phenotypic analysis, sexually mature male mice (6 weeks to 6 months old) were used. All mice were housed with a 12 h light cycle. All mouse experiments were performed according to the guidelines from the IACUC at Baylor College of Medicine (protocol AN-716).

### Reverse transcription-polymerase chain reaction (RT-PCR)

Mouse complementary DNA (cDNA) was cultivated from tissues of C57BL6J/129S5/SvEvBrd hybrid mice. Human multiple tissue cDNAs were purchased from BD bioscience. The following primers were used as performed [5]: Human *SSMEM1*, 5′–ATGAAGGCAGTGGGACAAG–3′ and 5′–CACTTCTGAGTTGGTTACAGGG; Human *GAPDH*, 5′–AATCCCATCACCATCTTCCAG–3′ and 5′–ATGACCCTTTTGGCTCCC–3′; mouse *Ssmem1*, 5′–AGCAAACAGGACGAAGACAG–3′ and 5′–TCGGTTACTGTGGAAACTTGG–3′; mouse *Hprt*, 5′–TGGATATGCCCTTGACTATAATGAG–3′ and 5′–TGGCAACATCAACAGGACTC–3′.

### Generation of *Ssmem1* KO mice

The pX330 plasmids expressing *hCas9* and single guide (sg)RNAs (ACAGATGTCTGAGAGCAAAC) targeting exon 2, which shares all splicing variants of *Ssmem1* were injected into pronuclei of zygotes [19]. Eggs were cultured in KSOM overnight and subsequently transferred into the oviducts of pseudopregnant Institute of Cancer Research (ICR) outbred female mice. Screening of mutant pups was performed by direct sequencing following polymerase chain reaction (PCR) using primers (5′–GCACTCATTTAACAGGGCTGA–3′ and 5′–GGTCTTTGCTGGCGTGATGA–3′). A founder mouse with a 6 bp deletion and 1 bp insertion was used to expand the colony. The genotyping was carried out by PCR with specific primers for the wild-type (WT) allele (primer a: 5′–ATGACTAGGGAGGAGCAGAGAC–3′ and primer b: 5′–ATTCCCATGACCACTCACTACC–3′) or KO allele (primer c: 5′–GACCACATCTTTCATGTCC–3′ and primer d: 5′–AGCAACTGAGAATGCAACCC–3′).

### Production of a monoclonal antibody against mouse SSMEM1

These procedures were performed as previously described [20]. In brief, the DNA sequence encoding mouse SSMEM1 (aa residues 58-224, ENSMUSG00000029784) with C-terminal 8xHis and 1D4 epitope tags were cloned into the pET15b (Novagen) plasmid vector. The plasmid was transformed into the Rosetta *Escherichia coli* strain (Millipore). The expression of SSMEM1-8xHis-1D4 protein was induced by adding isopropyl β-D-1-thiogalactopyranoside (IPTG) to a final concentration of 1.0 mM in LB medium. The cells were cultured at 30°C for overnight postinduction. After collection by centrifuge, the pellet was resuspended in Lysis buffer I [150 mM NaCl, 20 mM Tris-HCl pH 8.0, 10 mM Imidazole, 2% (v/v) Triton X-100, 1 mM DTT, 100 μg/ml Lysozyme, protease inhibitor cocktail tablets (Merck)] and lysed by ultrasonic disruptor (UD-201, TOMY). Triton-soluble fraction was removed by centrifugation (37 500 g, 30 min). The purified pellet (inclusion body) was resuspended in Lysis buffer II [150 mM NaCl, 20 mM Tris-HCl pH 8.0, 10 mM Imidazole, and 8 M Urea] and incubated overnight with gentle agitation. After centrifugation (37 500 g, 30 min), the supernatant was incubated with Ni-NTA Agarose (product no. 30210, QIAGEN) for 1 h with gentle agitation. The lysate was loaded on a column and washed with 40 ml of column wash buffer [150 mM NaCl, 20 mM Tris-HCl pH 8.0, 40 mM Imidazole, and 8 M Urea]. SSMEM1-8xHis-1D4 was eluted from the column with column elution buffer [150 mM NaCl, 20 mM Tris-HCl pH 8.0, 250 mM Imidazole, and 8 M Urea]. Recombinant SSMEM1 was used to produce the monoclonal antibody as previously described [21]. Specifically, purified SSMEM1 protein with Freund’s complete adjuvant was injected into female rats. After 17 days postinjection, lymphocytes were collected from iliac lymph nodes and hybridomas generated as described [22]. Supernatants from hybridoma cell cultures were collected and used as antibodies. The candidates were screened by the enzyme-linked immunosorbent assay against recombinant mouse SSMEM1.

### Western blot analysis

Testis and sperm were collected from adult mice and lysed in NP40 buffer (50 mM Tris-HCl pH 7.5, 150 mM NaCl, 0.5% NP40, and 10% Glycerol) followed by incubation for 1 h at 4°C with gentle agitation. The lysate was centrifuged at 15 000 g for 5 min at 4°C in a tabletop centrifuge. The supernatant was collected and subjected sodium dodecyl sulfate-polyacrylamide gel electrophoresis under reducing condition (with 5% 2-mercaptoethanol). The proteins from the gel were transferred to a polyvinylidene fluoride (PVDF) membrane. Membranes were blocked with 10% skim milk in Tris-buffered saline-Tween 20 (TBST; 20 mM Tris-HCl pH 7.6, 150 mM NaCl, and 0.1% Tween 20) for 1 h at room temperature (RT). Primary antibodies were diluted in blocking solution and incubated with the membranes overnight at 4°C. Membranes were washed three times with TBST before being incubated with antimouse IgG-HRP-conjugated secondary antibody for 1 h at RT. Membranes were then developed after washing. The blotted membrane was stained by Coomassie Brilliant Blue for a loading control.

### Histology

For histological analysis, testes were collected and fixed in Bouin’s fixative (Sigma Aldrich) for 3 h at RT. After several washes in 70% ethanol to remove excess stain, testes were embedded in paraffin. Tissues were sectioned at 5 μm thickness and stained by Periodic Acid-Schiff (PAS)-hematoxylin, followed by imaging with a 40× objective.

### Scanning electron microscopy (SEM)

Spermatozoa was recovered from cauda epididymis and placed in TYH medium (named after creators Toyoda, Yokoyama, and Hosi) at 37°C, 5% CO_2_ for 15 min [23]. Sperm were centrifuged at 300 × g for 5 min and washed in phosphate-buffered saline (PBS). After 2nd centrifuge, sperm were resuspended and fixed in 2.5% glutaraldehyde for 30 min at RT. Sperm were dehydrated in ethanol from 20% to 100%, incubating for 10 min in each. Sperm were transferred and resuspended in 50% t-butanol/50% ethanol for 15 min. The sperm samples were dried, and sputter coated with iridium, followed by imaging using Nova NanoSEM 230 at Houston Methodist Research Institute's Electron Microscopy Core.

### Sperm analysis

Sperm number and motility were measured as described previously [24]. Sperm were extracted by cutting caudal epididymis 30 times and incubated in human tubal fluid (HTF) media (Millipore) supplemented with 5% bovine serum albumin (BSA) under 5% CO_2_ at 37°C. After 15- or 90-min incubation, sperm samples were applied onto a chamber of 100 μm-depth analyzed counting slides (CellVision), and the sperm number and motility were measured using the Hamilton Thorne CEROS II system.

### Transmission electron microscopy (TEM)

Samples were prepared as previously described [25]. Epididymides and testes were quickly removed from sacrificed mice and fixed in 2% paraformaldehyde, 2.5% glutaraldehyde and 2 mM CaCl_2_ in 0.1 M cacodylate at 4°C overnight. After staining and dehydration, tissues were embedded in Spurr’s resin. Thin sections (80 nm) were stained with uranyl acetate and lead citrate, followed by observation using a Hitachi H7500 transmission electron microscope (Hitachi-HTA) at 80 kV at the Baylor College of Medicine Integrated Microscopy Core.

### Immunofluorescence

Testes were fixed in 4% paraformaldehyde (PFA)/PBS for overnight and replaced in from 10%, 20% to 30% sucrose at 4°C. The tissues were then embedded in OCT compound, freezing at −80°C. After sectioning at 10 μm, the samples were blocked with 3% BSA + 5% Normal Donkey Serum/PBS containing 0.1% Triton X-100 on the slides, for 1 h at RT. Primary antibodies were incubated with sections overnight at 4°C. Sections were washed with 3% BSA + 1% Normal Donkey Serum/PBS and incubated with secondary antibodies for 1 h at RT. 4′,6-diamidino-2-phenylindole (DAPI) was added in the washed buffer for 10 min in the final wash. Primary antibodies used were anti-Golgin 97 antibody at a concentration of 5 μg/ml (Abcam, #ab84340) and the lectin peanut agglutinin (PNA)-fluorescein isothiocyanate at a dilution of 1:500 (Sigma-Aldrich, #L7381). (Sigma). Secondary antibody was donkey antirabbit AF594 (Life Technologies). Slides were observed with Zeiss LSM 880 with Airyscan FAST Confocal Microscope at Baylor College of Medicine Optical Imaging & Vital Microscopy Core.

**Figure 1:**
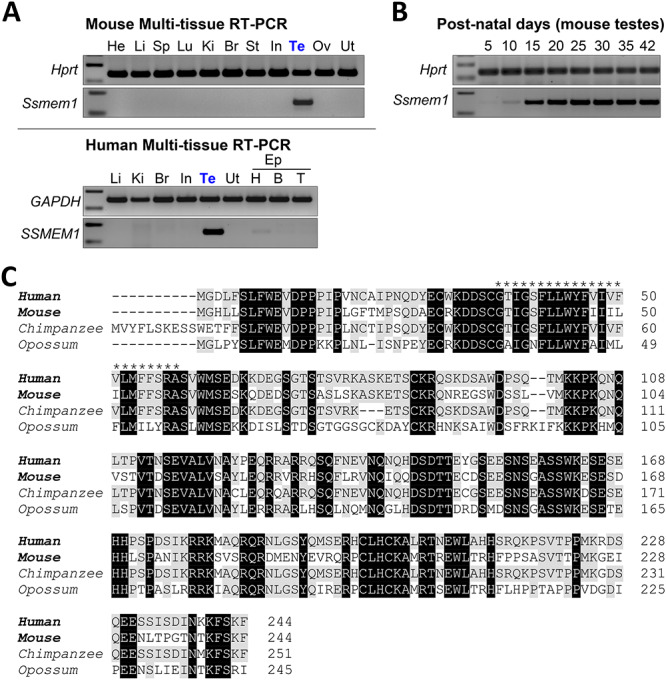
*Ssmem1* is a conserved testis-specific gene. (A) RT-PCR in multiple tissues confirming the testis specificity of *Ssmem1* in mice (Top panels) and humans (bottom panels). *Hprt* and *GAPDH* were used as controls. He, heart; Li, liver; Sp, spleen; Lu, lung; Ki, kidney; Br, brain; St, stomach; In, intestine; Te, testis; Ov, ovary; Ut, Uterus; Ep, Epididymis; H, head: B, Body; T, Tail. (B) RT-PCR from mouse testes at various postnatal days was performed. *Hprt* was used a control. (C) Protein sequence alignment of SSMEM1 from several mammalian species. Transmembrane region in human protein (asterisk) is well-conserved with other species. Black indicates fully conserved residues. Gray indicates conservation with residues in human.

### Statistical analysis

Statistical significance was evaluated by using the two-tailed unpaired Student *t*-test assuming unequal variances.

**Figure 2:**
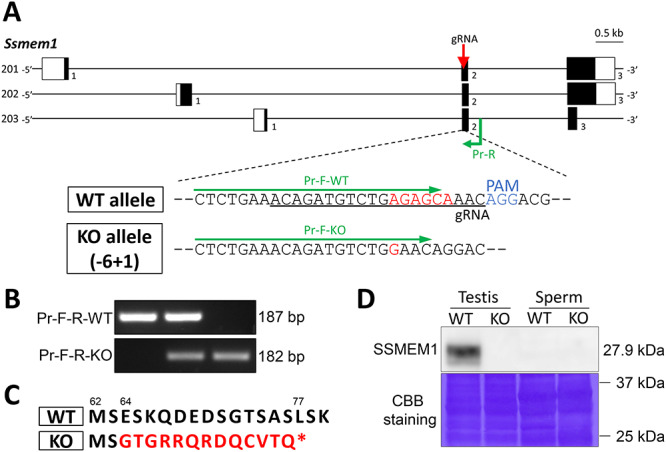
Generating *Ssmem1* KO mice. (A) Genomic structure of mouse *Ssmem1* and scheme to generate the gene KO mice using the CRISPR/Cas9 system. Three splice variants of *Ssmem1* have been reported. White and black boxes indicate untranslated and coding regions, respectively. Red arrow and black under bar indicate gRNA that targets the region. The delivery of CRISPR/Cas9 system into zygotes for mutagenesis via microinjection resulted in a 5 bp (6 bp deletion and 1 bp insertion) frameshift deletion (shown in red). (B) Genotyping of *Ssmem1* alleles. Primers indicated in green color in (A) amplify specific amplicon for the WT or KO allele. (C) Mutation in amino acid sequence in KO mouse induced by—five frameshift mutation is shown in red color. Asterisk indicates a premature stop codon. The numbers above sequences are amino acid number for each. (D) Western blot analysis using testis and sperm. SSMEM1 protein is detected only in WT testis. Equal loading of total protein was confirmed by Coomassie Brilliant Blue staining.

## Results

### *Ssmem1* is a highly conserved testis-specific gene

We identified *Ssmem1* as a conserved testis-specific gene by a bioinformatic screen as previously described [5]. We performed multitissue RT-PCR from male mice to reveal the *Ssmem1* expression profile. We obtained a single band in both human and mouse testes (Figure 1A). To determine the temporal expression pattern of *Ssmem1*, we performed RT-PCR using testis from WT mice of increasing age from postnatal day (PND) 5, 10, 15, 20, 25, 30, 35, and 42 to capture the earliest stage of expression of *Ssmem1* in mice. Expression of *Ssmem1* was observed at low levels at PND 5 and became pronounced at PND 15 (Figure 1B), which is coincident with the appearance of the first pachytene spermatocytes [26]. Protein sequence alignment analysis was performed on representative orthologs of SSMEM1 from different species. SSMEM1, which possesses a single transmembrane domain, is highly conserved in mammals, including human and mouse (Figure 1C).

**Figure 3:**
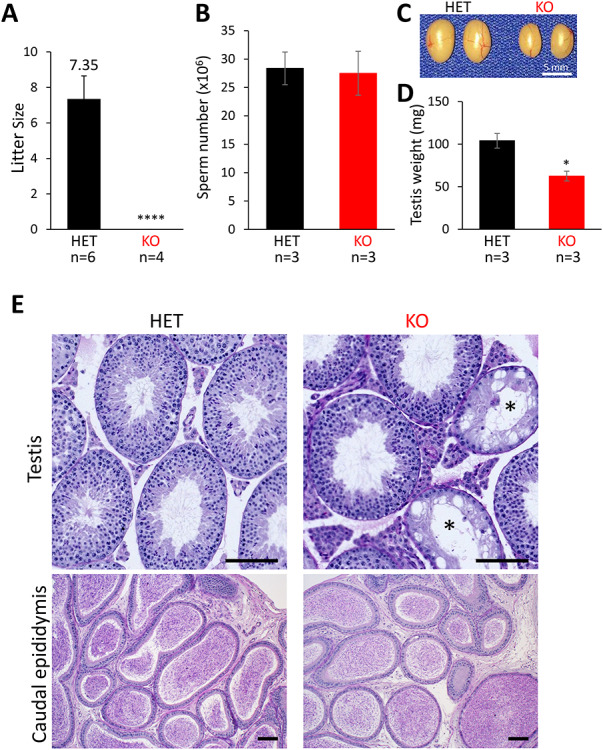
*Ssmem1* KO causes male infertility. (A) Average litter size from natural mating of *Ssmem1* HET and KO mice. Litter size was measured by the number of pups born. Ssmem1 KO males showed complete infertile, *P* < 0.0001. (B) Quantification of sperm released from the cauda epididymis. There was no significant difference between HET and KO mice. (C) Images of testes from *Ssmem1* HET and KO mice. (D) Average weight of individual testis. KO males showed a reduction in testis volume, *P* < 0.05. (E) PAS-Hematoxylin staining of testis and caudal epididymis sections from HET and KO mice. Occasional KO tubules in testis lacked germ cells (asterisks) (Scale bar, 100 μm)

### Generation of *Ssmem1* KO mice

To examine the physiological role of *Ssmem1*, we generated KO mice using the CRISPR/Cas9 system. The gRNAs were designed to target exon 2 which is shared with all *Ssmem1* splicing variants (Figure 2A). After an indel mutation containing a 6 bp deletion with a 1 bp insertion was confirmed by direct sequencing analysis, specific primers for WT or homozygous mutant (KO) allele were designed and used for genotyping (Figure 2B). The net 5 bp deletion of nucleotides, which caused a frameshift mutation, resulted in amino acid changes and a premature stop codon in KO mice in place of the L77 residue in WT mice (Figure 2C). We intercrossed the mouse line (−6 + 1 bp) to obtain subsequent generations and confirmed the absence of SSMEM1 protein in KO testes (Figure 2D). In addition, while SSMEM1 is present in testis, we found that the protein is absent from mature epididymal sperm from WT mice (Figure 2D). Observation of the KO mice revealed no obvious developmental abnormalities or differences in sexual behavior.

### *Ssmem1* is required for male fertility

Beginning at 6 weeks of age, sexually mature heterozygous or homozygous mutant males were housed with one female each for 6 months to test their fertility. The average number of offspring per litter and the number of litters were counted. Six control mating pairs had 7.3 ± 1.8 pups on average, whereas four homozygous mutant males exhibited sterility (Figure 3A). This data demonstrate that *Ssmem1* is essential for male fertility. To determine the cause of the sterility defect, we first analyzed the testis and sperm from controls and *Ssmem1* KO mice. The sperm count from caudal epididymis between HET and KO mice showed no significance difference (Figure 3B). However, the testicular size and weight from *Ssmem1* KO mice were significantly reduced compared to the control males (Figure 3C–D). In histological analysis of testes stained with PAS-hematoxylin, most tubules appeared normal but atrophic changes showing absence of germ cells and vacuolation in seminiferous tubules were occasionally observed in the *Ssmem1* KO (Figure 3E), which may explain the slight reduction in testis weight for the *Ssmem1* KO males. The epididymal histology showing the lumen filled with sperm in both HET and KO correlated with the results of the sperm count.

### *Ssmem1* KO results in spermiogenesis defects and motility reduction

**Figure 4:**
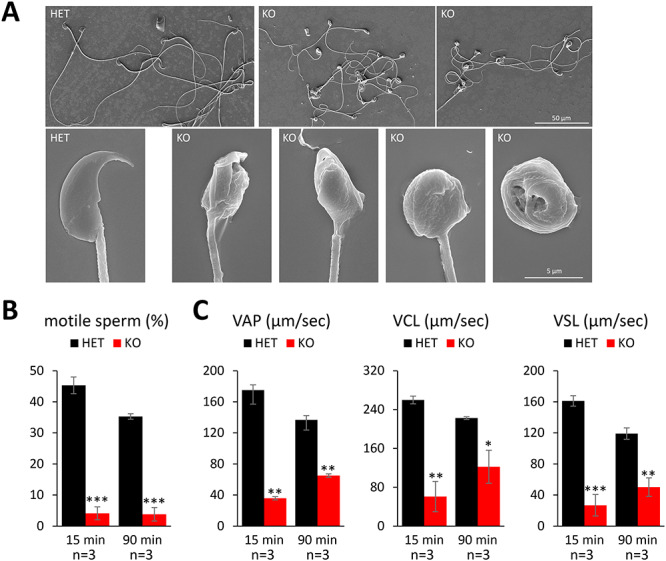
*Ssmem1* KO sperm show morphology and motility defects. (A) SEM images of HET/KO spermatozoa from caudal epididymis. KO sperm exhibited globozoospermia. (B) Sperm motility at 15 and 90 min after sperm suspension. There was a reduction in sperm motility in KO. (C) Sperm kinematic parameters measured using the CEROS II sperm analysis system. Velocities of KO sperm were lower than those of HET. *P* < 0.05 (^*^), *P* < 0.01 (^*^^*^), *P* < 0.001 (^*^^*^^*^).

To examine the morphology of spermatozoa, the sperm extracted from cauda epididymis were analyzed by SEM (Figure 4A). All KO spermatozoa showed a round or amorphous head morphology, and some presented with the tail coiled around the head (Figure 4A, bottom right). To further evaluate their infertility, we collected epididymal spermatozoa from adult *Ssmem1* HET and KO males and examined the sperm motility using a Computer Assisted Sperm Analysis (CASA) system. The percentage of motile sperm were significantly decreased in *Ssmem1* KO mice (4.1% at 15 min; 3.8% at 90 min incubation in sperm capacitation medium) compared to HET mice (45.3% at 15 min; 35.3% at 90 min; Figure 4B). In addition, motile sperm from KO mice showed impaired kinematic velocities after 90 min of incubation in capacitation medium (Figure 4C).

**Figure 5:**
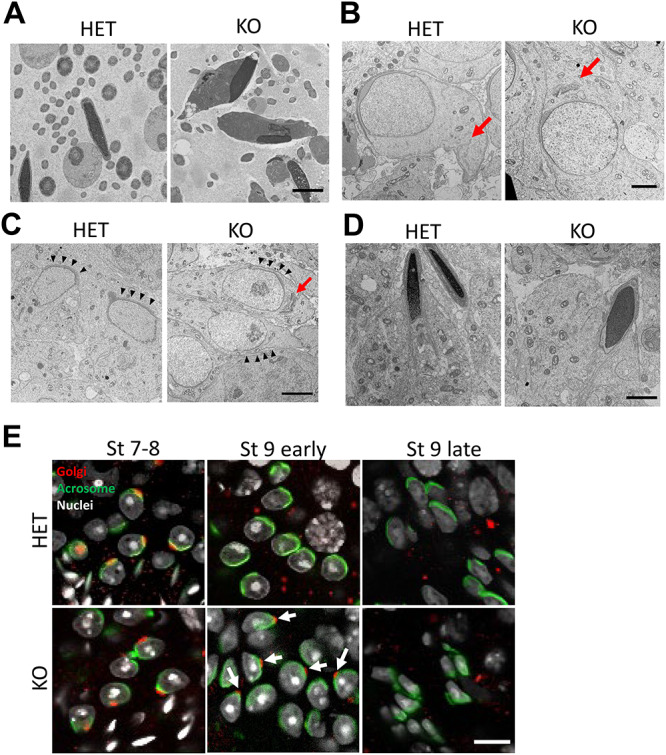
Ssmem1 KO caused a delay in organelle transfer. (A) TEM images of HET/KO spermatozoa in epididymis. Nuclei showed abnormal morphology and mitochondria are located at the sperm head region, alongside the nuclei (Scale bar, 2.0 μm). (B, C) TEM images of spermatids at same stages, step 8 (B); step 9 (C) in testis. In *Ssmem1* null spermatid, Golgi body (red arrows) failed to undergo migration as control spermatid at step 8. In step 9, elongating spermatids, the Golgi stayed in the caput aspect of the cell and the acrosomal membrane did not contact the cell surface (black arrowheads) in *Ssmem1* null spermatids (Scale bar, 4.0 μm). (D) TEM images of sperm at step 11. Golgi and mitochondria were located at caudal region of sperm in both HET and KO sperm (Scale bar, 2.0 μm). (E) Immunostaining of HET/KO testes. The Golgi body and acrosome were stained with Golgin 97 (red) and PNA (green), respectively. Arrows indicate the Golgi retained in acrosome in mutant spermatids. Migration of Golgi from acrosome side to tail side was delayed in KO spermatids (Scale bar, 10.0 μm).

### *Ssmem1* influences Golgi apparatus migration during spermiogenesis

The abnormalities of KO spermatogenesis were further examined by TEM. Epididymal observations revealed detailed abnormalities in the KO sperm: misshapen nuclei, disorganized mitochondria and tail entrapment in cytoplasmic droplets (Figure 5A). In HET testis, the Golgi in step 8 spermatids localized to the caudal region of the cell. In contrast, the Golgi failed to undergo migration to the caudal region in all spermatids at comparable stages in *Ssmem1* KO (Figure 5B); however, the formation of the acrosome did not appear to be disturbed. In step 9, elongating spermatids from HET controls, the Golgi and mitochondria completely migrated to the caudal side of the cell and the acrosomal membrane made contact with the cell plasma membrane (Figure 5C). In the KO spermatids, however, the Golgi remained in the rostral region of the cell and the acrosomal membrane did not attach to the plasma membrane. In step 11 spermatids (maturation phase), although the migration of the Golgi to the caudal side was finally observed in KO sperm, the shape of the nuclei and elongation of the cell were incomplete (Figure 5D). The abnormalities above were confirmed by immunofluorescent staining of testes, using GOLGIN-97 to label the trans-Golgi network and PNA to label the acrosome. The delayed migration of Golgi from acrosome side to tail side of the spermatid cytoplasm was observed in step 9 spermatids in KO testes (Figure 5E). These data suggest the delay of the Golgi migration during elongation of spermatids is defective during spermatogenesis in *Ssmem1* KO.

## Discussion

**Figure 6:**
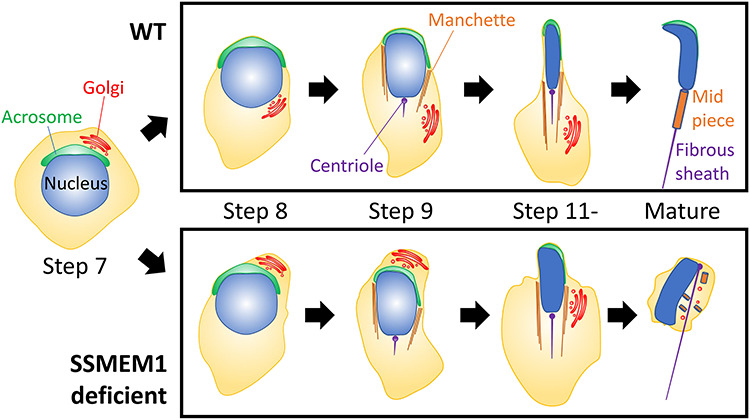
Schematic representation of Ssmem1 deficient spermatogenesis. The delay in organelle migration from step 8 to step 9 causes progressive changes leading to round-headed sperm in *Ssmem1* KO males.

By generating an *Ssmem1* KO model, we discovered that SSMEM1 is essential for male fertility. KO mice lacking *Ssmem1*, which is testis-specific and well-conserved in mammals, are sterile because of abnormal sperm head morphology (globozoospermia) and diminished motility. Absence of SSMEM1 causes a delay in intracellular repositioning of the Golgi during the transition from round spermatids to elongating spermatids in steps 7–9 of spermiogenesis. The Golgi is found lingering near the acrosome in step 9 spermatids of KO testes, which is followed by disrupted spermatid elongation, disorganization of cell organelles, derangement of sperm head morphology, and subsequent loss of sperm motility (Figure 6). These data imply that the mechanisms for spermatid elongation and for intracellular organelle trafficking might be independent, and that the organelle migration should occur before the initiation of elongation for appropriate spermatogenesis.

Studies of spermatogenesis have identified many acrosome-associated genes, such as *Zpbp1*, *Spaca1*, and *Spata46* as causes of globozoospermia [25, 27, 28]. Knockout of these genes in mice showed morphological defects of the acrosome during spermatogenesis, followed by disrupted sperm morphogenesis. In addition to the role of the mature acrosome in undergoing exocytosis (acrosome reaction) during transit through the female reproductive tract, the acrosome has a critical role in the development of proper sperm head shape [29, 30]. Unlike the previously mentioned genes essential for acrosome biogenesis, *Ssmem1* KO spermatids displayed normal acrosome formation in the early stages of spermatogenesis. In addition, all organelles were found to have normal morphology using ultrastructural analysis. The earliest phenotype in mouse spermatids lacking SSMEM1 is failure of the transfer of the Golgi from the rostral region of the polarized spermatid (near the acrosome) to the caudal region of the cell in step 7 and step 8 spermatids, when the spermatids begin elongation [31]. This abnormality is accompanied with the loss of the contact between the acrosomal membrane and the plasma membrane. Although the organelles eventually migrate to the caudal region of the cell for removal in KO testis at a later stage, the spermatids appear to have already lost the ability to orchestrate its organelles properly by that time, resulting in abnormal sperm morphology.

The localization and the molecular functions of SSMEM1 are not yet clear. Our RT-PCR analysis demonstrates that *Ssmem1* gene expression appears most drastically around PND 15, which corresponds to period in which cytoskeleton genes are expressed [32]. Furthermore, *spe-26,* which encodes a protein similar to actin-associated proteins, was reported in *C. elegans* where the *spe-26* mutant spermatocytes showed mislocalization of mitochondria, the endoplasmic reticulum (ER) and ribosomes which remained in the spermatid cytoplasm [33]. Similarly, myosin IV has been shown to be required for proper segregation of cell components including mitochondria and ER/Golgi-derived organelle complexes during *C. elegans* spermatogenesis [34]. In mice, intraflagellar transport protein 74 (IFT74)-deficient in testes caused a similar abnormal reorganization of cell organelles during spermatogenesis as in *Ssmem1* KO mice likely due to a failure of assembly of the cytoskeleton [35]. These data suggest SSMEM1 might associate with the cytoskeleton in spermatids. We hypothesize that SSMEM1, a transmembrane protein, localizes to the Golgi membrane and plays a role in the Golgi interaction with cytoskeletal proteins or associated proteins responsible for organelle migration during spermiogenesis. Other transmembrane proteins in the Golgi such as TM9SF3, TMED4/p25, and TMED7/p27 have been reported to be involved in Golgi migration after acrosome formation [36]. In addition, KO of TMF/ARA160 (TATA Element Modulatory Factor 1), which localizes to the Golgi in mouse testis and associates with tubulin and microtubules, demonstrate spermiogenesis failure attributable to disorientation of the Golgi and abnormal trafficking of the Golgi-derived proacrosomal vesicles during early spermiogenic steps [37]. Thus, we believe that SSMEM1 functions to anchor Golgi and cytoskeletal proteins for proper Golgi migration analogous to TMF/ARA160. Lastly, since SSMEM1 protein expression was only detected in the testis as shown in our western blot analysis (Figure 2D), it is likely that SSMEM1 is discarded into residual bodies and degraded with the Golgi before spermatids are released into the seminiferous tubule lumen during spermiation.

We show that generating KO mice using the CRISPR/Cas9 system is a powerful approach to explore implicit causative genes for male infertility. Future studies will investigate the subcellular localization of SSMEM1, and the interaction with IFT proteins and/or other cytoskeleton-associated proteins such as TMF. In addition, polymorphisms in human *SSMEM1* have been identified via the dbSNP database (www.ncbi.nlm.nih.gov/snp), suggesting that this gene could be contributing to human male infertility as well. The studies will have the potential for new insights into male infertility diagnosis and treatment as well as the development of nonhormonal contraceptives.
